# Physiological, morphological hair, and blood parameters of the goat group genetic Azul in the semiarid

**DOI:** 10.1007/s00484-026-03195-4

**Published:** 2026-04-30

**Authors:** Jaciara Ribeiro Miranda, Roberto Germano Costa, Maria Norma Ribeiro, Edilson Paes Saraiva, Valquíria Cordeiro da Silva, Neila Lidiany Ribeiro

**Affiliations:** 1 Physiological, morphological hair, and blood parameters of the goat group genetic Azul in the semiarid, Areia, PB Brazil; 2 Departamento de Zootecnia, Centro de Ciências Agrárias, Universidade Federal da Paraíba - UFPB, Areia, Paraíba Brasil; 3 Departamento de Zootecnia, Universidade Federal Rural de Pernambuco – UFRPE, Pernambuco – PE Recife, Brazil; 4 Universidade Federal de Campina Grande-UFCG, Campina Grande, Paraíba, Brasil

**Keywords:** Adaptability, Cortisol, Local breeds, Rectal temperature, Thyroid

## Abstract

This study was conducted to investigate the effects of the climatic seasons on physiological, morphological, and hormonal parameters in female goats of the Brazilian Azul genetic group. Thirty females were used in the rainy season and thirty in the dry season, all non-lactating and non-pregnant, aged between 1 and 2 years. The data were analyzed using the Statistical Analysis System (SAS) and the General Linear Models procedure. Air temperature, black globe temperature, and relative humidity were measured by an automated weather station. Respiratory rate (RR), heart rate (HR), and surface temperature (TS) showed significant differences between periods and seasons, whereas rectal temperature did not vary. Animals in the rainy season had longer hair compared to the dry season, with a significant difference (*P* = 0.0258). In the erythrogram, significant seasonal differences were observed for all variables analyzed: hematocrit (*P* = 0.0258), hemoglobin (*P* = 0.0365), red blood cell count (*P* = 0.0269), mean corpuscular volume (*P* = 0.0258), and mean corpuscular hemoglobin concentration (*P* = 0.0157).The animals had a lower respiratory rate and greater heat dissipation capacity during the dry season, allowing them to maintain rectal temperature. Morphological and hormonal (T3, T4, and cortisol) parameters change across the various climatic seasons.

**Supplementary Information:**

The online version contains supplementary material available at 10.1007/s00484-026-03195-4.

## Introduction

The hardiness and good adaptation of native goats make them an interesting genetic resource for semi-arid conditions. Similar adaptive strategies have been reported globally, with Mediterranean, Asian, and desert breeds showing resilience to water scarcity, heat stress, and limited forage availability (Silanikove, 1992; Caroprese et al., 2012; Geldsetzer-Mendoza and Riveros 2023; Rodrigues et al., 2023; Ribeiro et al. 2026). These findings reinforce the interpretation that goats worldwide exhibit convergent adaptive mechanisms, underscoring their role in sustainable livestock management under climate change.

Through natural selection over several generations, goats have acquired a high survival capacity, exhibiting common characteristics such as small size, short hair, and small, straight ears, which are differentiated by hair color. These characteristics support the interpretation that the data reflect adaptive mechanisms in arid and semiarid environments. Small ruminants are homeothermic animals and are characterized by the ability to maintain body temperature within narrow limits, using for this purpose exchanges with the environment, internal heat production, morphological, physiological, and hormonal characteristics (Morais et al., 2024; Geldsetzer-Mendoza and Riveros 2023).

Morphological characteristics: hair density, hair diameter and length, and skin color are factors that affect the effectiveness of heat loss by evaporation in animals. Differences in anatomical and morphological characteristics can partially explain the differences in heat tolerance between species and breeds (Fonseca et al., 2024; Morais et al., 2024; Mascarenhas et al., 2023). When the animal is subjected to stressful environmental conditions, its physiological functions, rectal temperature, respiratory rate, heart rate, and surface temperature, as well as hormonal and morphological parameters, are altered (Fonseca et al., 2024).

Some studies demonstrate greater concern with the animal x environment relationship, caused by an environment due to the joint action of variables, such as temperature, humidity and radiation so that the animal remains in thermal comfort, that is, not suffering thermal stress, since there is relative knowledge between heat stress and productivity, in intensive and extensive breeding systems (Cardoso et al. 2021a, b, 2023; Fonseca et al., 2024; Geldsetzer-Mendoza and Riveros 2023). Therefore, the objective was to study the physiological, morphological, and hormonal parameters in female goats of the Brazilian Azul genetic group, while accounting for the effect of the climatic season.

## Materials and methods

### Local experiment

The study was conducted in the municipality of Caiçara do Rio do Vento, located in the state of Rio Grande do Norte, Brazil (5°45’37” S and 35°59’55” W). Based on the Koppen climate classification, the average annual temperature over the years is 27.2 °C, with a range of 21.0 –33.0 °C.

### Managements and animals

This research received approval from the Animal Ethics Committee of the Federal University of Paraíba (UFPB) under protocol number 6167/18.

Thirty goats, all of them non-lactating and non-pregnant females belonging to the Blue genetic group, were evaluated. The animals’ ages were indirectly estimated by dental chronometry, and all were classified as adults (2 years old).

Animals were screened for ectoparasites, lymphadenitis, and other skin problems, and dewormed. The females were kept in a free-range grazing environment on native pasture (lowland caatinga) and had unrestricted access to a shelter.

### Thermal comfort índices and climatological data

**Fig. 1:**
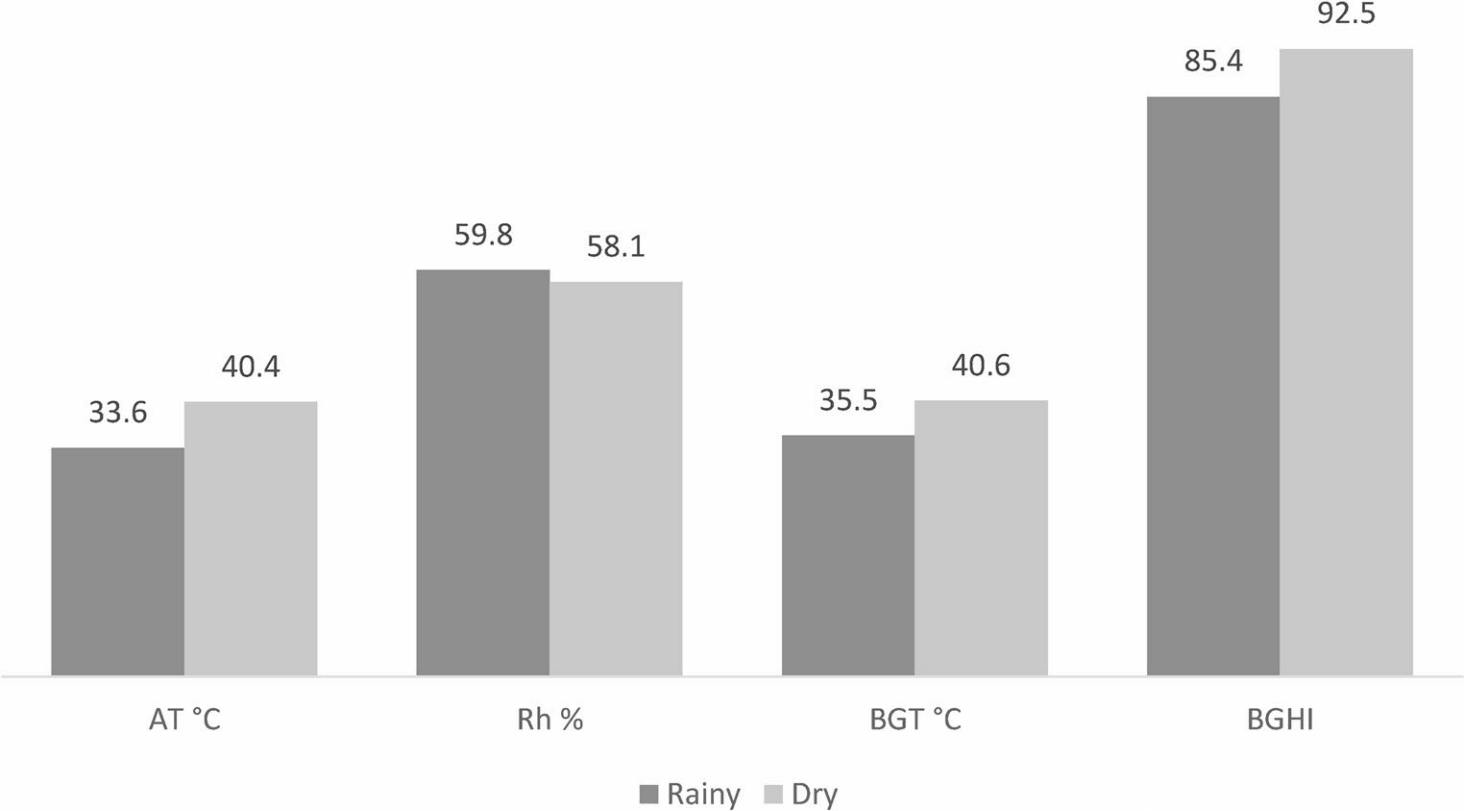
Temperature values ​​(AT), relative humidity (RH), black globe temperature (BGT) and black globe temperature and humidity index (BGHI) in the rainy and dry season

Thermological data were also collected on the data collection days using an automatic weather station that was located with the animals during the day. Every 2 h, a digital anemometer was used to measure wind velocity. Using the black globe temperature (Tbg) and the dew point temperature (Tdp), the black globe and humidity index (BGHI) was calculated according to Buffington et al. (1981). The black globe temperature and relative humidity were recorded at 15-second intervals. The environments in these two seasons (winter and summer) were characterized using averages of the climatological data (Fig. 1).

### Physiological parameters

In both seasons, physiological parameters were collected over three consecutive days, in the morning (from 08:00) and in the afternoon (from 14:00). A veterinary clinical thermometer (32.0–43.9 °C) was used to measure the rectal temperature (RT) of the animals by inserting it in the rectum of each animal, with its bulb directly contacting the mucosa. Respiratory rate (RR) and heart rate (HR) were evaluated by indirect auscultation over the first ribs in the right thoracic region with a stethoscope, counting samples for 20 s; the obtained value was multiplied by 3 to calculate the total number of **breaths** per minute and the total number of beats per minute. The surface temperature (ST) was recorded with a Minipar MT-350 digital infrared thermometer (Shanghai/China) at a distance of 50 cm from the body, measuring the left flank.

### Anatomical parameters

From each animal, hair was manually collected from the central lateral trunk region once per season. The hairs were placed in paper envelopes according to their identification for assessment, with the aim of noting their morphological categorization by length and diameter. Average hair length (µm) was calculated as the mean of the ten largest hairs measured with a digital caliper, Udo (1978), and average diameter was determined using a digital micrometer (Digital 50, DIGIMESS), calibrated to 0.001 mm.

### Blood samples

Blood samples were taken from each animal once every season in the afternoon (15:00 h) by puncturing the jugular vein after disinfecting with iodine alcohol.

For evaluating hematological parameters, blood was collected in 5-ml vacuum tubes containing 10% anticoagulant ethylenediaminetetraacetic acid (EDTA) (Jain, 1993).

Blood was collected in 7-ml vacuum tubes containing separate gel and sodium fluoride (for glucose analysis) and centrifuged afterwards in a digital centrifuge at 4 °C and 3000 rpm (1100XG) for 15 min for biochemical and hormonal parameters analysis. After centrifugation, the supernatant was divided into 1.5-mL aliquots for biochemical and hormonal assays, which were conducted the day after collection. Plasma samples were stored at -20 °C prior to the assay (Cardoso et al. 2023; Rodrigues et al., 2023).

A range of parameters was evaluated, including total protein (TP), albumin (ALB), glucose (GLU), triglycerides (TRI), cholesterol (CHO), urea (URE), creatinine (CRE), gamma-glutamyl transferase (GGT), aspartate aminotransferase (AST), and alanine aminotransferase (ALT). These analyses were performed using a biochemical analysis instrument (Thermo Scientific Genesys 10 S Vis, Centreville, VA, USA) equipped with a multi-wavelength photometer, employing commercially available kits (Labtest).

For the quantification of cortisol (COR), total thyroxine (T4), and total triiodothyronine (T3), we used a microplate absorbance spectrophotometer (BIO-RAD xMark, Hercules, CA) to perform duplicate measurements using a competitive enzyme-linked immunosorbent assay (ELISA). Hormone quantification was conducted using kits from In Vitro Diagnostic Ltda., Itabira, Brazil.

### Statistical analyses

This study adopted a fully randomized design with a 2 × 2 factorial of 2 seasons (rainy and dry) and 2 periods of the day (morning and afternoon). ANOVA was used to compare means using the Student’s t-test at the 5% level, using the GLM procedure in SAS On Demand (2024).

Pearson’s correlation coefficients among all variables were estimated using the CORR procedures of SAS On Demand (2024). The number of components was determined from the eigenvalues, using the Kaiser criterion (1960) apud Mardia (1979), i.e., only components with eigenvalues greater than 1 were retained. Analyses were conducted using the Statistica software (version 8.0).

## Results

Respiratory rate (RR), heart rate (HR), and surface temperature (TS) showed significant differences between periods and seasons, whereas rectal temperature did not vary (Table 1). Specifically, rectal temperature did not show significant differences (*P* = 0.4582 for season; *P* = 0.5847 for period; *P* = 0.4859 for season*period interaction). RR was significantly higher in the afternoon and during the dry season (*P* = 0.0258 for season; *P* = 0.0158 for period; *P* = 0.0242 for interaction). HR also varied significantly, with higher values in the dry season and in the afternoon (*P* = 0.0365 for season; *P* = 0.0264 for period; *P* = 0.0325 for interaction). TS showed marked differences, being higher in the dry season and in the afternoon (*P* = 0.0258 for season; *P* = 0.0025 for period; *P* = 0.0052 for interaction). This variation is associated with oscillations in climatic factors, indicating that environmental variables were outside the thermal comfort zone.

**Table 1:** Mean ± standard deviation of physiological variables in goats of the azul genetic group across dry and rainy seasons, measured in the morning and afternoon

Season	Period	Rectal temperature (°C)	Respiratory rate (breaths min^− 1^)	Heart rate (beat min^− 1^)	Surface temperature (°C)
Rainy	Morning	39.28 ± 0.36a	27.16 ± 6.49 d	78.47 ± 11.50d	35.49 ± 0.34 d
	Afternoon	39.54 ± 0.40a	36.50 ± 5.09 c	83.25 ± 15.66c	37.49 ± 1.25 c
	Mean	39.41 ± 0.40 A	31.83 ± 7.80 B	80.86 ± 13.00B	36.49 ± 1.5 B
Dry	Morning	39.32 ± 0.56a	38.95 ± 8.58 b	92.57 ± 20.57b	39.94 ± 2.10 b
	Afternoon	39.53 ± 0.40a	39.58 ± 10.44 a	94.75 ± 16.79a	40.79 ± 1.19 a
	Mean	39.43 ± 0.60 A	39.27 ± 10.500 A	93.68 ± 12.00 A	40.38 ± 2.6 A
*P-value*					
Season		0.4582	0.0258	0.0365	0.0258
Period		0.5847	0.0158	0.0264	0.0025
Season*period		0.4859	0.0242	0.0325	0.0052

^a, b,c, d^ Different letters in the column differ from each other by the t-test (*P* < 0.01)

^A, B^ Different letters in the column differ from each other by the t-test (*P* < 0.01)

Animals in the rainy season had longer hair compared to the dry season, with a significant difference (*P* = 0.0258) (Table 2). Hair thickness was also greater in the rainy season compared to the dry season, with a significant difference (*P* = 0.0058). Hair diameter was smaller during the rainy season and larger during the dry season, with a significant difference (*P* = 0.0369). The number of hairs per cm² did not differ significantly between seasons (*P* = 0.0582). These characteristics indicate that, during the rainy season, longer, thinner hair favors heat retention, while during the dry season, shorter, thicker hair facilitates air circulation and heat dissipation.

**Table 2:** Mean ± standard deviation of hair morphological parameters in female goats of the azul genetic group

Variable	Season		*P*-value
	Rainy	Dry	
Hair length (cm)	4.20 ± 1.20 a	3.08 ± 0.50 b	0.0258
Hair thickness (mm)	0.85 ± 0.05 a	0.65 ± 0.06 b	0.0058
Hair diameter (mm)	0.07 ± 0.01 a	0.08 ± 0.01 b	0.0369
Number of hairs (hairs/cm^2^)	1799.70 ± 207.90a	1734.56 ± 205.70a	0.0582

^a, b^Different letters in the line differ from each other by the t-test (*P* < 0.01)

In the erythrogram, significant seasonal differences were observed for all variables analyzed: hematocrit (*P* = 0.0258), hemoglobin (*P* = 0.0365), red blood cell count (*P* = 0.0269), mean corpuscular volume (*P* = 0.0258), and mean corpuscular hemoglobin concentration (*P* = 0.0157) (Table 3). For the biochemical profile, significant differences were found in glucose (*P* = 0.0365), cholesterol (*P* = 0.0352), triglycerides (*P* = 0.0152), urea (*P* = 0.0258), creatinine (*P* = 0.0485), total protein (*P* = 0.0025), albumin (*P* = 0.0125), globulin (*P* = 0.0058), GGT (*P* = 0.0142), AST (*P* = 0.0035), and ALT (*P* = 0.0152).

**Table 3:** Mean ± standard deviation of erythrogram, blood biochemical, and hormonal parameters in azul goats during rainy and dry seasons in the brazilian semiarid

Variables	Rainy	Dry	P-value
Erythrogram			
Hct %	40.14 ± 2.60a	24.12 ± 6.21b	0.0258
Haemoglobin g/dL	15.28 ± 1.62a	12.00 ± 2.80b	0.0365
RBC x 10^6^ mL	18.22 ± 1.92a	15.70 ± 3.50b	0.0269
MCV f/L	24.98 ± 2.09a	10.82 ± 3.11b	0.0258
CHCM g/dL	32.91 ± 0.21a	30.61 ± 0.30b	0.0157
Blood biochemical			
Glucose mg/dL	120.18 ± 4.80a	100.94 ± 5.72b	0.0365
Cholesterol mg/dL	190.56 ± 29.80a	162.61 ± 35.63b	0.0352
Triglycerides mg/dL	14.25 ± 2.96b	17.58 ± 3.95a	0.0152
Urea mg/dL	72.13 ± 10.06b	74.10 ± 12.24a	0.0258
Creatinine mg/dL	1.20 ± 0.29a	1.12 ± 0.33b	0.0485
Total Protein g/dL	4.68 ± 0.65b	7.37 ± 0.88a	0.0025
Albumin g/dL	1.98 ± 0.45b	2.86 ± 0.49a	0.0125
Globulin g/dL	2.71 ± 0.58b	4.49 ± 1.01a	0.0058
GGT U/L	49.21 ± 10.11a	41.61 ± 9.14b	0.0142
AST U/L	82.18 ± 14.20a	71.89 ± 13.81b	0.0035
ALT U/L	70.20 ± 15.14b	95.42 ± 9.32a	0.0152
Hormonal			
T4 µg/mL	1.54 ± 0.19a	0.99 ± 0.29b	0.0120
T3 µg/mL	1.89 ± 0.23a	1.00 ± 0.20b	0.0389
Cortisol ng/mL	3.29 ± 0.90b	5.80 ± 1.29a	0.0147

^a,b^Different letters in the line differ from each other by the t-test (P<0.01)

*Hct* haematocrit, *RBC* erythrocytes, *MCV* mean corpuscular volume, *MCHC* mean corpuscular haemoglobin concentration, gamma glutamyl transferase SL (*GGT*), aspartate aminotransferase (*AST*) and alanine amino transferase (*ALT*); total thyroxine (*T4*) and total triiodothyronine (*T3*)

In the hormonal profile, significant differences were observed for T4 (*P* = 0.0120), T3 (*P* = 0.0389), and cortisol (*P* = 0.0147). During the rainy season, characterized by lower temperatures and higher relative humidity (Fig. 1), T3 and T4 concentrations were higher. Conversely, the highest mean cortisol levels were recorded during the dry season (*P* < 0.01), which influenced blood cortisol concentrations (Table 3). Cortisol secretion is a physiological response to stress, including increased air temperature during the summer (Fig. 1).

Even after data standardization, three components were required to meet the selection criterion (eigenvalue ≥ 1.0), together accounting for nearly 65% of the accumulated variance (Table 4). Among the 11 variables studied, Hemoglobin, Mean Globular Volume, ST, RR, HR, and hair diameter showed the strongest correlations with the first three principal components.

**Table 4:** Eigenvalues, percentage of variance, and correlations (factor loading) of each variable and its respective principal component (PC)

Variables	PC1	PC2	PC3
Hematocrit	**0.930**	-0.035	-0.019
Mean red blood cell volume	**0.920**	-0.044	-0.199
Hemoglobin	0.601	-0.283	0.098
Erythrocytes	0.615	-0.165	0.224
Hair length	0.584	-0.468	0.402
Hair diameter	-0.183	-0.384	**0.701**
Hair thickness	-0.473	-0.313	0.535
Rectal temperature	0.057	-0.563	-0.486
Respiratory rate	-0.198	**-0.698**	-0.271
Heart rate	-0.265	**-0.755**	-0.276
Surface temperature	**-0.728**	-0.129	-0.071
Eigenvalue	3.694	1.971	1.434
Cumulative variation	33.588	51.509	64.548

PC1 (Hemoglobin, Mean Globular Volume, ST): PC1 reflects the balance between hematological parameters and thermoregulation. As ST decreases, hemoglobin concentration and mean globular volume increase. This suggests that during the dry season, dehydration leads to hemoconcentration, elevating hematocrit and mean corpuscular volume. The higher blood concentration improves oxygen transport but simultaneously requires heat dissipation through the body surface. Thus, PC1 captures the trade-off between blood physiology and thermal regulation.

PC2 (Respiratory Rate, Heart Rate): PC2 is defined by respiratory and cardiac activity. Interestingly, these variables did not increase alongside rectal temperature, indicating that goats may rely less on elevated respiratory or cardiac rates to cope with heat. This stability suggests a physiological tolerance to climatic stress, minimizing energy expenditure while maintaining homeothermy.

PC3 (Hair Diameter): PC3 is represented by hair diameter, which varies with seasonal changes. Increased hair diameter during the dry season may act as a morphological adjustment to modulate heat exchange, without significantly affecting rectal temperature, respiratory rate, heart rate, or surface temperature. This highlights the role of integumentary traits in adaptation.

Overall, the increase in erythrocytes, hemoglobin, hair length, mean globular volume, rectal temperature, and hematocrit was accompanied by decreases in ST, hair thickness, hair diameter, RR, and HR. This inverse relationship underscores adaptive mechanisms: hematological adjustments and integumentary traits work together to maintain rectal temperature within narrow limits, despite environmental stress.

**Fig. 2:**
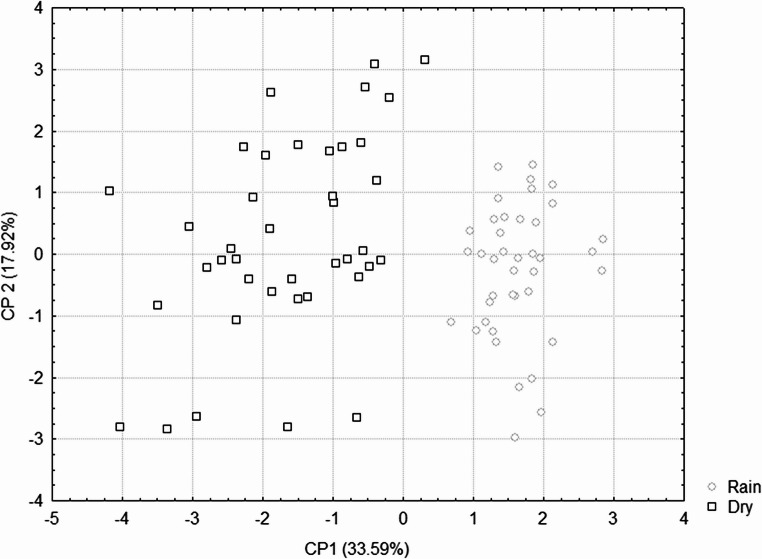
Principal Component Analysis (PCA) of physiological, morphological, and hormonal parameters in Azul goats during rainy and dry seasons

The x-axis (CP1, 33.59% of variance) and y-axis (CP2, 17.92% of variance) represent the two principal components that explain the largest proportion of variability in the dataset (Fig. 2). Each point corresponds to an individual goat, with circles indicating animals sampled in the rainy season and squares representing those sampled in the dry season. It can be observed that in the dry season, animals employ adaptation mechanisms in diverse ways, resulting in a more dispersed distribution, whereas in the rainy season, the same animals exhibit similar adaptive responses, forming a more homogeneous group. This separation along CP1 highlights the influence of seasonal conditions on biological responses and demonstrates distinct adaptive profiles under different climatic environments.

Discriminant analysis further supported these findings: hemoglobin and heart rate were excluded because they did not contribute to distinguishing individuals, while the discriminant function derived from the remaining variables correctly classified 100% of the goats. This confirms the PCA grouping, where individuals are clearly separated by season. The classification function was expressed as: y = − 10.274 + 22.780⋅Mean Globular Volume + 4.145⋅Hair Size − 8.801⋅Hematocrit + 2.047⋅Erythrocyte − 1.353⋅Hair Thickness + 1.192⋅Rectal Temperature − 1.208⋅Surface Temperature − 0.691⋅Hair Diameter.

Together, the PCA and discriminant analysis provide strong evidence of seasonal influences on adaptive mechanisms in Azul goats, reinforcing their distinct physiological and morphological profiles in the semiarid environment.

## Discussion

It is common in semiarid regions during the hottest part of the day for the temperature to remain above the thermal comfort zone (Cardoso et al. 2021a, b, 2023). Native goats have shown good productive performance (Cardoso et al. 2023; Ribeiro et al. 2026), due to the adaptive process to the semiarid region, developed throughout their formation, even in conditions considered above the comfort zone, an extremely positive fact for the breed and indicative of the need to redefine the adaptability parameters for native breeds of the semiarid Northeast. The black globe temperature and humidity index values ​​should not be considered as a dangerous situation for the goat breeds studied, because although there are no reference values ​​for local goats, these values ​​cannot be considered dangerous, since the RT is within the normal range, demonstrating that there is no heat storage.

Goats are active during the day, which alters their physiological parameters. The daily variation of RT during the day was 0.26 °C in the rainy season and 0.21 °C in the dry season. According to Piccione and Refinetti (2003), this variation can be from 0.30 °C to 1.90 °C. The physiological parameters showed higher values in the afternoon and during the dry season. Physiological responses increase according to air temperature (Fonseca et al., 2024; Matos Júnior et al. 2021; Cardoso et al. 2021a, b, 2023).

Rectal temperature remained within the limits for the goat species, indicating that the animals used heat-dissipation mechanisms effectively, as evidenced by increased RR and ST. Analyzing the relationship between season and time of day reveals that an animal’s adaptability can be evaluated by its capacity to respond to both typical environmental conditions and extreme weather, while sustaining or experiencing minimal decline in productive output. In the research, this adaptability was measured by the animal’s ability to regulate its body temperature after sun exposure, using mechanisms for heat dissipation (Santos et al., 2023). In this study, to maintain RT within physiological limits, the animals increased RR without entering thermal stress. According to Silanikove (2000), goats remain in low stress at RR values up to 40 breaths per minute, and in this study, RR values remained within that threshold. Thus, the increase in RR should be interpreted as a functional thermoregulatory response rather than as evidence of pathological stress.

In tropical climates, animals should ideally have light-colored fur, with short, thick, well-set hair under a highly pigmented epidermis. Mascarenhas et al. (2023) state that the amount of radiation effectively transmitted through the fur layer depends not only on the color but also on the degree of its physical structure, especially the number of hairs per unit area. Hematological parameters are altered in animals to maintain core temperature within the thermal comfort zone, within the range recommended for the species. RBC values ​​are adjusted so that the animal survives both food and water shortages and high temperatures (40 °C in the dry season). The values obtained from this study are consistent with previous reports (Kaneko et al., 2009; Cardos et al., 2023; Ribeiro et al. 2026). These changes are adaptive and were acquired over the years through selection that enabled the animal to survive in the region. At very high temperatures, as is the case during the dry season (40 °C), animals increase glucose production and mobilize triglycerides to produce energy, and catabolism slows down, as there will be less creatinine in the bloodstream.

Thyroid hormones (inverse correlation [-0.55, *P* < 0.05]) are inversely correlated with air temperature and maintain acceptable limits of rectal temperature in animals according to standard species criteria. The thyroid hormone levels were higher during the rainy season, when temperatures decreased. Physiological responses to environmental changes are rapid in animals well adapted to their environments (Rodrigues et al., 2023; Fonseca et al., 2024). According to Rodrigues et al. (2023), this decline in hormone concentration acts as a regulatory mechanism to attenuate heat. There is an increase in cortisol concentration, which helps maintain the animal’s homeostasis. Goats subjected to heat stress (32 °C) presented 6.44 ng mL^− 1^ for cortisol, 1.23 µg dL^− 1^ for T3, and 1.97 µg dL^− 1^ for T4; these animals in thermal comfort (26 °C) presented 5.86 ng mL^− 1^ for cortisol, 1.56 µg dL^− 1^ for T3, and 2.11 µg dL^− 1^ for T4 (Cardoso et al. 2021a).

Cortisol shows a direct correlation with air temperature (0.89, *P* < 0.05) and with thyroid hormones; the correlations are inverse, being − 0.64 (*P* < 0.05) with T4 and 0.58 (*P* < 0.05) with T3. There is an inverse relationship between thyroid hormone and AT concentrations in goats, an adaptive mechanism that reduces heat production. The T4 hormone presents an inverse correlation with air temperature (-0.65 *P* < 0.05), and the T3 hormone also presents the same behavior with air temperature (-0.67 *P* < 0.05). Cortisol presents an inverse correlation with the length of the animal’s hair (-0.55, *P* < 0.05). As the season becomes hotter, the animal’s hair changes in size to aid in adaptation. Cortisol showed a direct correlation with surface temperature (0.65, *P* < 0.05), whereas T4 showed an inverse correlation (-0.58, *P* < 0.05). This is because, as air temperature increases, the surface temperature rises to dissipate heat, and the increase in cortisol is also due to the rise in air temperature, while T4 decreases to reduce endogenous heat production.

In both seasons of our experiment, the air temperature was between 34 and 43 °C, well above the air temperature used by Cardoso et al. (2021a) in their experiment when evaluating native goats, where they found low concentrations for the hormones T3 and T4 of 32 °C, 1.23 µg dL^− 1^ and 1.97 ngmL^− 1^, respectively, while the cortisol concentration increased at a temperature of 32 °C (6.44 ngmL^− 1^), a behavior similar to the concentrations found for the Azul goat. Physiological variables are good indicators of animal health, but they must be properly interpreted. There is greater classification error when considering only physiological variables, which is reduced when considering more than one group (Correa et al., 2013). This is well accounted for in the equation mentioned above, which considers groups of physiological and anatomical variables.

## Conclusions

The animals had a lower respiratory rate and greater heat dissipation capacity during the dry season, allowing them to maintain rectal temperature. Morphological and hormonal (T3, T4, and cortisol) parameters change across the various climatic seasons. Rectal temperature and respiratory rate cannot fully characterize the animals’ adaptability. In the absence of rectal temperature changes, the animals employ erythrocyte mechanisms and increased surface temperature to dissipate heat.

## Supplementary Information

Below is the link to the electronic supplementary material.

Supplementary Material 1.

## Data Availability

Not applicable.
