# Supplementary material for: Physiological, morphological hair, and blood parameters of the goat group genetic Azul in the semiarid
Source: Int J Biometeorol. 2026 Apr 30;70(5):150. doi: 10.1007/s00484-026-03195-4 (PMC13133218; doi:10.1007/s00484-026-03195-4)
Supplement: Supplementary file 1 — Supplementary Material 1. [file 484_2026_3195_MOESM1_ESM.pdf]

justdone

Scan details

Scan Time:	Total Pages:	Total Words:
March 30th, 2025 at 11:35 UTC	18	4367

Analysis Report

Plagiarism Detection Report

JustDone AI

Plagiarism Detection

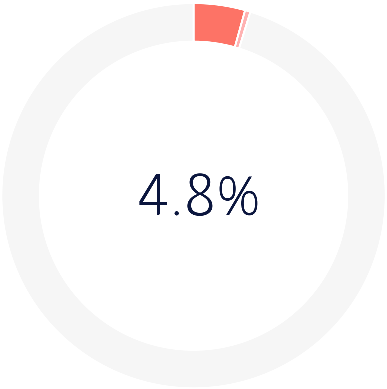

Plagiarism Types	Text Coverage	Words
Identical	4.3%	189
Minor Changes	0.5%	20
**Excluded**		
Omitted Words		0

Certified by
Copyleaks

About this report
help.copyleaks.com

Plagiarism

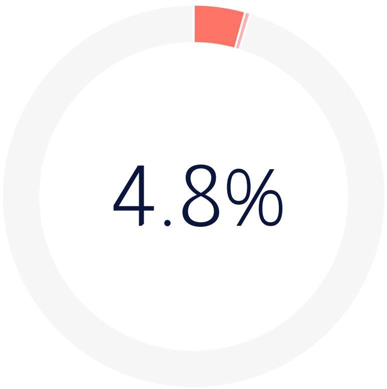

Results (36)

**Repository**<br>N/A	**Internal Database**<br>0
**Internet Sources**<br>36	**Current Batch**<br>0

Plagiarism Types	Text Coverage	Words
Identical	4.3%	189
Minor Changes	0.5%	20
**Excluded**		
Omitted Words		0

About Plagiarism Detection

Our AI-powered plagiarism scans offer three layers of text similarity detection: Identical, Minor Changes, and Paraphrased. Based on your scan settings we also provide insight on how much of the text you are not scanning for plagiarism (Omitted words).

Identical

One to one exact word matches. Learn more

Minor Changes

Words that hold nearly the same meaning but have a change to their form (e.g. "large" becomes "largely"). Learn more

Omitted Words

The portion of text that is not being scanned for plagiarism based on the scan settings. (e.g. the 'Ignore quotations' setting is enabled and the document is 20% quotations making the omitted words percentage 20%) Learn more

Copyleaks Internal Database

Our Internal Database is a collection of millions of user-submitted documents that you can utilize as a scan resource and choose whether or not you would like to submit the file you are scanning into the Internal Database. Learn more

Filtered and Excluded Results

The report will generate a complete list of results. There is always the option to exclude specific results that are not relevant. Note, by unchecking certain results, the similarity percentage may change. Learn more

Current Batch Results

These are the results displayed from the collection, or batch, of files uploaded for a scan at the same time. Learn more

Plagiarism Detection Results: (36)

(PDF) Effects of the dry and the rainy season on endocrine and physiologic pr... 1.7%

https://www.researchgate.net/publication/321585282_effects_of_the_dry_and_the_rainy_season_on_endocrine_a...

ArticlePDF AvailableEffects of the dry and the rainy season on endocrine and physiologic profiles of goats in the Brazilian semi-ari...

(PDF) Goats in a comfortable and stressed environment consuming saline water:... 1.2%

https://www.researchgate.net/publication/374360854_goats_in_a_comfortable_and_stressed_environment_con...

ArticlePDF AvailableGoats in a comfortable and stressed environment consuming saline water: performance,digestibility,nitrogen...

?format=pdf&lang=en 1.1%

https://www.scielo.br/j/abmvz/a/djn7cnccx8w7lbcgdk9fcmd/?format=pdf&lang=en

Betty

http://dx.doi.org/10.1590/1678-4162-12804 Arq.Bras.Med.Vet.Zootec.,v.75,n.5,p.967-974,2023 Goats in a comfortable and stressed ...

Effects of the dry and the rainy season on endocrine and physiologic profiles... 1.1%

https://flore.unifi.it/retrieve/e398c37c-8fc0-179a-e053-3705fe0a4cff/effects%20of%20the%20dry%20and%2...

Ribeiro Neila Lidiany

Italian Journal of Animal Science ISSN: (Print) 1828-051X (Online) Journal homepage: http://www.tandfonline.com/loi/tjas20 Effects of t...

SciELO Brazil - Goats in a comfortable and stressed environment consuming sal... 1.1%

https://www.scielo.br/j/abmvz/a/djn7cnccx8w7lbcgdk9fcmd/?format=html&lang=en

...

SciELO Brazil - Goats in a comfortable and stressed environment consuming sal... 1.1%

https://www.scielo.br/j/abmvz/a/djn7cnccx8w7lbcgdk9fcmd/?format=html&lang=en

...

Adaptive profile of Garfagnina goat breed assessed through physiological, hae... 1.1%

https://www.researchgate.net/publication/308923310_adaptive_profile_of_garfagnina_goat_breed_assessed_thr...

Home Eutheria Goats ArticleAdaptive profile of Garfagnina goat breed assessed through physiological,haematological,bioc...

(PDF) Biochemical and hormonal parameters of goats kept in a controlled envir... 1%

https://www.researchgate.net/publication/354078986_biochemical_and_hormonal_parameters_of_goats_kept_in...

ArticlePDF AvailableBiochemical and hormonal parameters of goats kept in a controlled environment consuming water with different...

Biochemical and hormonal parameters of goats kept in a controlled environment... 0.6%

https://www.scienceopen.com/document?vid=7066639f-f62b-4b06-96fd-7c8de9a2617f

ScienceOpen: research and publishing network For PublishersDiscoveryMetadataPeer reviewHostingPublishing For ResearchersJoinPublishRev...

Biochemical and hormonal parameters of goats kept in a controlled environment... 0.6%

https://doaj.org/article/cddc8755db0045c2975bc7d1858ab4aa

This website uses cookies to ensure you get the best experience. Learn more about DOAJ's privacy policy. Hide t...

Heat stress in horses: a literature review - PMC 0.6%

https://pmc.ncbi.nlm.nih.gov/articles/pmc10267279/

Skip to main content...

Mechanisms and evolution of resistance to environmental extremes in animals -... 0.6%

https://pmc.ncbi.nlm.nih.gov/articles/pmc6862762/

Skip to main content...

Laser and thermographic infrared temperatures associated with heat tolerance ... 0.5%

https://www.alice.cnptia.embrapa.br/alice/bitstream/doc/1030727/1/1s2.0s0921448815300081xmain.pdf

C.A.Cruz Júnior

Small Ruminant Research 132 (2015) 86-91 Contents lists available at ScienceDirect Small Ruminant Research journal homepage: www.elsevi...

SombraModificaRespostas.pdf 0.5%

https://repositorio.ufu.br/bitstream/123456789/34103/3/sombramodificarespostas.pdf

UNIVERSIDADE FEDERAL DE UBERLÂNDIA FACULDADE DE MEDICINA VETERINÁRIA NATANI SILVA REIS SOMBRA MODIFICA AS RESPOSTAS COMPORTAMENTAIS E F...

Shade Modifies Behavioral and Physiological Responses of Low to Medium Produc... 0.5%

https://pmc.ncbi.nlm.nih.gov/articles/pmc8388728/

Skip to main content...

(PDF) Physiological parameters for thermal stress in dairy cattle 0.5%

https://www.researchgate.net/publication/308243166_physiological_parameters_for_thermal_stress_in_dairy_cat...

ArticlePDF AvailablePhysiological parameters for thermal stress in dairy cattle August 2016 Revista Brasileira de Zootecnia...

001081609.pdf?sequence=1&isAllowed=y 0.5%

https://lume.ufrgs.br/bitstream/handle/10183/186087/001081609.pdf?sequence=1&isallowed=y

Revista Brasileira de Zootecnia © 2016 Sociedade Brasileira de Zootecnia ISSN 1806-9290 www.sbz.org.br R.Bras.Zootec.,45(8):458-465,...

(PDF) Physiological and biochemical blood variables of goats subjected to hea... 0.4%

https://www.researchgate.net/publication/323963507_physiological_and_biochemical_blood_variables_of_goats...

Home Eutheria Goats ArticlePDF AvailablePhysiological and biochemical blood variables of goats subjected to heat stress - a rev...

SciELO Brazil - Goats in a comfortable and stressed environment consuming sal... 0.4%

https://www.scielo.br/j/abmvz/a/djn7cnccx8w7lbcgdk9fcmd/abstract/?lang=en

...

SciELO Brazil - Biochemical and hormonal parameters of goats kept in a contro... 0.4%

https://www.scielo.br/j/abmvz/a/xqccw46v7tvxtdkpsjkhgfs/

...

-Water balance of Yankasa sheep and Madari goat in North Nigeria | Download T... 0.4%

https://www.researchgate.net/figure/water-balance-of-yankasa-sheep-and-madari-goat-in-north-nigeria_tbl1_2...

Table 1 - uploaded by Sílvia TurcoContent may be subject to copyright.Download View publication Copy reference Copy caption Embed...

Heat tolerance of goats to increased daily maximum temperature and low salini... 0.4%

https://pmc.ncbi.nlm.nih.gov/articles/pmc11065954/

Skip to main content...

?lang=en&format=pdf 0.4%

https://www.scielo.br/j/abmvz/a/xqccw46v7tvxtdkpsjkhgfs/?lang=en&format=pdf

Evaldo

http://dx.doi.org/10.1590/1678-4162-12186 Arq.Bras.Med.Vet.Zootec.,v.73,n.4,p.853-860,2021 Biochemical and hormonal parameters o...

Rumin-D-15-7359R2.pdf 0.4%

https://flore.unifi.it/bitstream/2158/1069138/1/rumin-d-15-7359r2.pdf

Elsevier Editorial System(tm) for Small Ruminant Research Manuscript Draft Manuscript Number: Rumin-D-15-7359R2 Title: Adaptive profile o...

SciELO Brazil - Physiological parameters for thermal stress in dairy cattle P... 0.3%

https://www.scielo.br/j/rbz/a/sjfh36bz8dvxq9qwm4dvfzg/

...

Mean (±SEM) values of respiratory rate of T4 in adult bucks of RSG and... | D... 0.3%

https://www.researchgate.net/figure/mean-sem-values-of-respiratory-rate-of-t4-in-adult-bucks-of-rsg-and-sh...

Figure - available from: Journal of Applied Animal ResearchThis content is subject to copyright. Terms and conditions apply....

Mean (±SEM) values of T3:T4 in bucks of RSG and SHG during the... | Download ... 0.3%

https://www.researchgate.net/figure/mean-sem-values-of-t3t4-in-bucks-of-rsg-and-shg-during-the-cold-dry-...

Figure - available from: Journal of Applied Animal ResearchThis content is subject to copyright. Terms and conditions apply....

s41389276_phd_final.pdf?Expires=1743420287&Key-Pair-Id=APKAJKNBJ4MJBJNC6NLQ&S... 0.3%

https://espace.library.uq.edu.au/data/uq_380155/s41389276_phd_final.pdf?expires=1743420287&key-pair-id=ap...

Angela Lees

Biological Responses of Feedlot Cattle to Heat Load Angela Maree Lees B.App Sc.(Hons) A thesis submitted for the degree of Doctor of P...

PII: S0301-6226(00)00162-7 0.3%

http://publicationslist.org.s3.amazonaws.com/data/silanikove/ref-59/lps.welfareheatstress.review.pdf

Livestock Production Science 67 (2000) 1-18 www.elsevier.com / locate / livprodsci Review article Effects of heat stress on the welfare...

(PDF) Temperature-Humidity Indices as Indicators to Heat Stress of Climatic C... 0.3%

https://www.researchgate.net/publication/329232536_temperature-humidity_indices_as_indicators_to_heat_stre...

Home Animals, Domestic ArticlePDF AvailableTemperature-Humidity Indices as Indicators to Heat Stress of Climatic Conditions...

Metabolic and hormonal acclimation to heat stress in domesticated ruminants 0.3%

https://dspace.unitus.it/bitstream/2067/1536/1/final%20animal%20repo_a77rkgly.pdf

U. Bernabucci, N. Lacetera, L. H. Baumgard, R. P. Rhoads, B. Ronchi, A. Nardone

animal Animal (2010), 4:7, pp 1167-1183 & The Animal Consortium 2010 doi:10.1017/S175173111000090X Metabolic and hormonal acclimation t...

General Systemic States - PMC 0.3%

https://pmc.ncbi.nlm.nih.gov/articles/pmc7195945/

Skip to main content...

(PDF) Metabolic and hormonal acclimation to heat stress in domesticated rumin... 0.3%

https://www.researchgate.net/publication/221973396_metabolic_and_hormonal_acclimation_to_heat_stress_in_d...

ArticlePDF AvailableMetabolic and hormonal acclimation to heat stress in domesticated ruminants July 2010 animal 4(7):1167-83 D...

(PDF) Effect of the environment and diet on the physiological variables of sh... 0.3%

https://www.researchgate.net/publication/332922967_effect_of_the_environment_and_diet_on_the_physiologic...

Nayanne Lopes Batista Dantas

ArticlePDF AvailableEffect of the environment and diet on the physiological variables of sheep in the Brazilian semi-arid region April...

---

vol2023_2.pdf 0.3%

https://animalsciencejournal.usamv.ro/pdf/2023/issue_2/vol2023_2.pdf

SCIENTIFIC PAPERS SERIES D.ANIMAL SCIENCE Volume LXVI,No.2 BucharesT 2023 Faculty of Animal Productions Engineering and Management...

Heat Stress and Goat Welfare: Adaptation and Production Considerations - PMC 0.3%

https://pmc.ncbi.nlm.nih.gov/articles/pmc8065958/

Skip to main content...

Physiological, morphological hair and blood parameters of goat group genetic A zul in semiarid

Jaciara Ribeiro Miranda1, Roberto Germano Costa 2 , Maria Norma Ribeiro 3 , Edilson Paes Saraiva 2 , Valquíria Cordeiro da Silva 4 , Neila Lidiany Ribeiro 2 *, 1Dados do TCC realizado na Universidade Federal da Paraiba - UFPB, Areia/Paraiba/Brasil 2 Departamento de Zootecnia. Universidade Federal da Paraíba - UFPB, Centro de Ciências Agrárias, Areia-Paraíba-Brasil 3 Departamento de Zootecnia. Universidade Federal Rural de Pernambuco - UFRPE, Recife - Pernambuco - PE 4 Universidade Federal de Campina Grande-UFCG, Campina Grande - Paraíba - Brasil * Autor para correspondência: neilalr@hotmail.com

Abstract This study was carried out to investigate the effect of the climatic seasons on physiological, morphological and hormonal parameters in female goats of the Brazilian Azul genetic group. Thirty females were used in the rainy season and thirty in the dry season, all non-lactating and non-pregnant, aged between 1 and 2 years. The data was analyzed using the Statistical Analysis System, applying the general linear models procedure. Air temperature, black globe temperature and relative humidity were measured by an automated weather station. The values for physiological parameters (rectal temperature, respiratory rate, heart rate and surface temperature) were higher in the afternoon (P<0.05) in both seasons of the dry season, being 39.34°C, 39.58 mov./ min, 94.75 bat./min and 40.79 °C, respectively. For the anatomical parameters (hair diameter, length and thickness), there was a significant effect of season (P<0.05), with the values of 0.07µm, 4.20 cm and 0.85 cm being the highest in the rainy season. There was a significant effect (P<0.05) for hormones as a function of the effect of the climatic season on hormonal parameters. The animals' ability to adapt cannot be described exclusively by rectal temperature and respiratory rate.

Keywords: Adaptability. Cortisol. Local breeds. Rectal temperature. Thyroid

Introduction The hardiness and good adaptation of native goats make them interesting genetic material for semi-arid conditions, as they are animals raised predominantly under extensive management, tolerate low forage availability, water scarcity, along with high temperatures and intense solar radiation (Rodrigues et al., 2023). Through a process of natural selection over several generations, goats have acquired a high survival capacity, presenting common characteristics such as: small size, short hair, small and straight ears, differentiated between them by the color of the hair. These characteristics help the animals adapt to arid and semiarid regions. Small ruminants are homeothermic animals and are characterized by the ability to maintain body temperature within narrow limits, using for this purpose exchanges with the environment, internal heat production, morphological, physiological and hormonal characteristics (Morais et al., 2024) Morphological characteristics: hair density, hair diameter and length, skin color, are factors that affect the effectiveness of heat loss by evaporation in animals. Differences in anatomical and morphological characteristics can partially explain the differences in heat tolerance between species and breeds (Fonseca et al., 2024; Morais et al., 2024; Mascarenhas et al., 2023). When the animal is subjected to stressful environmental conditions, its physiological functions, rectal temperature, respiratory rate, heart rate and surface temperature as well as hormonal and morphological parameters are altered (Fonseca et al., 2024). Some studies demonstrate greater concern with the animal x environment relationship, caused by an environment due to the joint action of variables, such as temperature, humidity and radiation so that the animal remains in thermal comfort, that is, not suffering thermal stress, since there is relative knowledge between heat stress and productivity, in intensive and extensive breeding systems (Cardoso et al., 2021a, 2021b; 2023; Fonseca et al., 2024). Therefore, the objective was to study the physiological, morphological and hormonal parameters in female goats of the Brazilian genetic group Azul considering the effect of the climatic season. Material and methods Local experiment The study was conducted in the municipality of Caiçara do Rio do Vento, located in the state of Rio Grande do Norte, Brazil (5°45'37' S and 35°59'55' W). Based on the Koppen climate classification, the average annual temperature over the years is 27.2°C, with a range of 21.0°C - 33.0°C. Managements and a nimals This research received approval from the Animal Ethics Committee of the Federal University of Paraíba (UFPB) under protocol number 6167/18 . Thirty goats, all of them non-lactating and non-pregnant females belonging to the Blue genetic group, were evaluated. The age of the animals was indirectly estimated by dental chronometry, and all of them were classified as adults (2 years). Animals were screened for

ectoparasites, lymphadenitis, or other skin problems and were dewormed. The females were kept in a free-range grazing environment on native pasture (lowland caatinga) and had unrestricted access to a shelter. Thermal comfort índices and c limatological data Thermological data were also collected on the data collection days using an automatic weather station that was pooled with the animals during the day. Every 2 hours, a digital anemometer was used to measure wind velocity. Using the black globe temperature (Tbg) and the dew point temperature (Tdp), the black globe and humidity index (BGHI) was calculated according to Buffington et al. (1981). The b lack globe temperature and relative humidity were recorded at 15-second intervals . The environment in these two seasons (winter, summer) was characterized using the averages of the climatological data (Figure 1). Physiological parameters In both seasons, physiological parameters were collected over three consecutive days, in the morning (from 08:00) and in the afternoon (from 14:00). A veterinary clinical thermometer (32.0°C–43.9°C) was used to measure the rectal temperature (RT) of the animals by inserting it in the rectum of each animal, with its bulb directly contacting the mucosa. Respiratory rate (RR) and heart rate (HR) were evaluated by indirect auscultation over the first ribs in the right thoracic region with a stethoscope, counting samples for 20 seconds; the obtained value was multiplied by 3 to calculate the total number of movements per minute and the total number of beats per minute. Th e surface temperature (S T) was recorded with a Minipar MT-350 digital infrared thermometer (Shanghai/China) at 50 cm distance from the body measuring the left flank. Anatomical parameters From each animal, hair was manually collected in the centrolateral region of the trunk once per season. The hairs were placed in paper envelopes according to their identification for assessment to note the morphological categorization of hair in reference to length and diameter. Average hair length (µm) was calculated as a mean of the ten largest hairs measured with a digital caliper, Udo (1978), and average diameter was determined using a digital micrometer (Digital 50, DIGIMESS), calibrated to 0.001 mm. Blood samples Blood samples were taken from each animal once every season in the afternoon (15:00 h) by puncturing the jugular vein after disinfecting with iodine alcohol . For evaluating hematological parameters, blood was collected in 5-ml vacuum tubes with 10% of anticoagulant ethylene diamine tetra acetic acid (EDTA) (Jain, 1993). Blood was collected in 7-ml vacuum tubes containing separate gel and sodium fluoride (for glucose analysis) and centrifuged afterwards in a digital centrifuge at 4°C and 3000 rpm (1100XG) for 15 minutes for biochemical and hormonal parameters analysis. After centrifugation, the supernatant was divided into 1.5-mL aliquots for biochemical and hormonal assays; the assays were conducted the day after the collection. Plasma samples were stored at -20°C prior to the assay (Cardoso et al., 2023; Rodrigues et al., 2023). A range of parameters were evaluated, including total protein (TP), albumin (ALB), glucose (GLU), triglycerides (TRI), cholesterol (CHO), urea (URE), creatinine (CRE), gamma-glutamyl transferase (GGT), aspartate aminotransferase (AST), and alanine aminotransferase (ALT). These analyses were performed using a biochemical analysis instrument (Thermo Scientific Genesys 10S Vis, Centreville, VA, USA) equipped with a multi-wavelength photometer, employing commercially available kits (Labtest). For the quantification of cortisol (COR), total thyroxine (T4), and total triiodothyronine (T3) concentrations, we used a microplate absorbance spectrophotometer (BIO RAD xMark, Hercules, CA) to carry out duplicate measurements via a competitive enzyme-linked immunosorbent assay (ELISA). Hormone quantification was conducted using kits from In Vitro Diagnostic Ltda., Itabira, Brazil Statistical analyses This study adopted a fully randomized design with a 2x2 factorial of 2 seasons (rainy and dry) and 2 periods of the day (morning and afternoon). ANOVA was used for comparing means by the Student's test at the 5% level, using the GLM procedure of SAS On Demand (2024). Pearson's correlation coefficients among all variables were estimated using the CORR procedures of SAS OnDemand (2024). The number of components was determined from the eigenvalues, considering the Kaiser criterion (1960) apud Mardia (1979), meaning that only those components whose eigenvalues were higher than 1 were considered. Analyses were conducted using the Statistica software (version 8.0) Results Respiratory rate (RR), heart rate (HR) and surface temperature (TS) were significantly different (P<0.0 5) between periods and seasons (Table 1), while rectal temepratude did not show any significant difference. The animals' TS showed a significant difference (P>0.0 5 between seasons and periods (Table 1). TS showed a higher value in the afternoon and in the dry season. This variation in TS is associated with the oscillation of climatic factors, and the environmental variables were outside the thermal comfort zone. Respiratory rate (P<0.0 5) was higher in the afternoon and during the dry season. A high RR does not mean that the animal is under heat stress, as it is another

thermoregulation parameter . Animals in the rainy season have thicker fur (P<0.05), which is useful for protecting animals from excessive wind (Table 2). In the rainy season, the fur is longer and has a smaller diameter in order to retain hot air for warmth. In the dry season, the fur is shorter and has a larger diameter, thus allowing air circulation and cooling the skin. In the dry season, the fur thickness was thinner to facilitate heat dissipation (Table 2). The red blood series of goats presented higher values in the rainy season (P<0.05) (Table 3). Glucose, cholesterol, creatinine, GGT, AST, T3 and T4 also presented higher concentrations in the rainy season (P<0.05) (Table 3), evidencing the influence of the joint action of meteorological variables (Figure 1) on the endocrine and thermoregulatory physiology of the animals. A decline in plasma levels of T3 and T4 was observed in the dry season. In the rainy season, with low temperatures and relative humidity (Figure 1), the concentrations of T3 and T4 were higher. The highest mean cortisol was recorded in the dry season (P<0.01), which influenced the blood concentration of cortisol (Table 3). Cortisol secretion occurs as a response to stress, with the increase in air temperature in the summer (Figure 1) . Even with data standardization, three components were needed to meet the selection criterion adopted (>=1.0), with almost 65% of the accumulated variance (Table 4). Thus, the nature and degree of relationship of the variables are decisive in defining the number of factors to be selected. Among the 11 variables studied, only Hemoglobin, Mean Globular Volume, ST, RR, HR and hair diameter were the variables with the highest correlation with the first three principal components. The first principal component (PC1) is represented by the variables Hemoglobin, mean globular volume and ST. It can be observed that as ST decreases, the variables Hemoglobin and mean globular volume increase. The hematocrit value is higher in the dry season, as hemoconcentration occurs due to dehydration, consequently increasing the mean globular volume. In this way, the animal dissipates heat through the surface temperature. The second principal component (PC2) is represented by the variables respiratory and heart rate. These do not increase, nor does RT increase, possibly showing greater tolerance to climate changes. The third principal component (PC3) is represented by the variable hair diameter. With the change in season, the hair diameter changes to help the animal adapt. There was an increase in hair diameter, but it did not affect RT, RR, HR and ST. This shows that the animals are adapted to the environmental conditions, as they used mechanisms to adapt the animal without changing the rectal temperature. The increase in the variables erythrocytes, hemoglobin, hair length, mean globular volume, RT and hematocrit is followed by a decrease in the variables ST, hair thickness, hair diameter, RR and HR. At high temperatures, the hematocrit, erythrocyte, mean globular volume, hair size and RT increase to adapt the animal organism to that situation, however, as these variables increase, others decrease their value, this is the case of the variables ST, hair thickness, hair diameter, RR and HR, showing that these animals are adapted to the environment. When the season changes, changes occur in the hair diameter and thickness. It can be seen in figure 2 that in the dry season, animals use adaptation mechanisms in a variety of ways, showing themselves to be quite dispersed, while in the rainy season these same animals use adaptation mechanisms in a similar way, showing themselves to be a more homogeneous group. Based on the formation of the groups, it can be concluded that the individuals had variations in their adaptation characteristics. In the discriminant analysis, the variables hemoglobin and heart rate were discarded because they did not contribute to the discrimination of individuals. The discriminant function determined by the other variables allowed for the adequate classification of 100% of the individuals, confirming what was seen in Figure 2, in which the individuals can be seen adequately grouped in their group of origin. The classification function of individuals to their respective groups (season) was: y = -10.274 + 22.780 mean globular volume + 4.145 hair size -8.801 hematocrit +2.047 erythrocyte -1.353 hair thickness + 1.192 rectal temperature -1.208 surface temperature -0.691 hair diameter. Discussion It is common in semiarid regions, during the hottest times of the day, for the temperature to remain above the thermal comfort zone (Cardoso et al., 2021a, b; 2023). Native goats have shown good productive performance (Cardoso et al., 2023), due to the adaptive process to the semiarid region, developed throughout its formation, even in conditions considered above the comfort zone, an extremely positive fact for the breed and indicative of the need to redefine the adaptability parameters for native breeds of the semiarid Northeast. The black globe temperature and humidity index values should not be considered as a dangerous situation for the goat breeds studied, because although there are no reference values for local goats, these values cannot be considered dangerous, since the RT is within the normal range, demonstrating that there is no heat storage . Goats are active during the day, which causes changes in their physiological parameters. The daily variation of RT during the day

was 0.26oC in the rainy season and 0.21oC in the dry season. According to Piccione and Refinetti (2003), this variation can be from 0.30oC to 1.90oC. The physiological parameters presented higher values in the afternoon and in the dry season. Physiological responses increase according to air temperature (Fonseca et al., 2024; Matos Júnior et al., 2021; Cardoso et al., 2021 a,b ; 2023). R ectal temperature remained within the limit for the goat species, indicating that the animals used heat dissipation mechanisms effectively, with an increase in RR and ST. Analyzing the relationship betwe en season and time of day reveals that an animal's adaptability can be evaluated by its capacity to respond to both typical environmental conditions and extreme weather, while sustaining or experiencing minimal decline in productive output. In the research conducted, this adaptability was measured by the animal's ability to regulate its body temperature following sun exposure, utilizing mechanisms for heat dissipation (Santos et al., 2023). In this study, to maintain RT within the physiological limit, the animals increased RR, but remained out of thermal stress; according to Silanikove (2000), up to 40 mov/min the animal is in low stress. The HR presented a higher mean value (P<0.01) in the dry season due to the fact that at high temperatures peripheral vasodilation and a decrease in blood pressure occur to reduce heat production. However, if the animals remain under thermal stress due to heat, an increase in HR occurs. In tropical climates, animals should ideally have light-colored fur, with short, thick, well-set hair under a highly pigmented epidermis. Mascarenhas et al. (2023) state that the amount of radiation effectively transmitted through the fur layer depends not only on the color but also on the degree of its physical structure, especially the number of hairs per unit area. Hematological parameters are altered by animals as a way of maintaining a core temperature within the thermal comfort zone within the range recommended for the species. RBC values are adjusted so that the animal survives both food and water shortages and high temperatures (40°C in the dry season). The values obtained from this study are consistent with previous reports (Kaneko et al. 2009; Cardos et al., 2023). These changes are adaptive and were acquired over the years as a result of the selection that the animal underwent to survive in the region. At very high temperatures, as is the case during the dry season (40°C), animals increase glucose production and mobilize triglycerides to produce energy, and catabolism slows down, as there will be less creatinine in the bloodstream. Thyroid hormones (inverse correlation [-0.55, P<0.05]) are inversely correlated with air temperature and maintain acceptable limits of rectal temperature in animals according to standard species criteria. The thyroid hormones showed higher values during the rainy season when there was a decrease in temperature. Physiological responses to environmental changes are rapid in animals well adapted to their environments (Rodrigues et al., 2023; Fonseca et al., 2024). According to Rodrigues et al. (2023), these hormones concentration decline acts as a regulatory mechanism to attenuate heat. There is an increase in the concentration of cortisol, which has the function of maintaining the animal's homeostasis. Goats subjected to heat stress (32 °C) presented 6.44 ng mL-1 for cortisol, 1.23 µg dL-1 for T3 and 1.97 µg dL-1 for T4; these animals in thermal comfort (26 °C) presented 5.86 ng mL-1 for cortisol, 1.56 µg dL-1 for T3 and 2.11 µg dL-1 for T4 (Cardoso et al., 2021a). Cortisol has a direct correlation with air temperature (0.89, P<0.05), and with thyroid hormones the correlation is inverse, being -0.64 (P<0.05) with T4 and 0.58 (P<0.05) with T3. There is an inverse relationship between the concentrations of thyroid hormones and A T in goats, being an adaptive mechanism to reduce heat production. The T4 hormone presents an inverse correlation with air temperature (-0.65 P<0.05) and the T3 hormone also presents the same behavior with air temperature (-0.67 P<0.05). Cortisol presents an inverse correlation with the length of the animal's hair (-0.55 P<0.05), as the season of the year becomes hotter the animal's hair changes in size to aid in adaptation. Cortisol presented a direct correlation with surface temperature (0.65 P<0.05), contrary to what occurred with T4 where its correlation with surface temperature is inverse (-0.58 P<0.05). This is due to the fact that as the air temperature increases, the surface temperature also increases to dissipate heat, and the increase in cortisol is also due to the increase in air temperature, while T4 decreases to reduce endogenous heat production . In both seasons of our experiment, the air temperature was between 34 and 43 °C, well above the air temperature used by Cardoso et al. (2021a) in their experiment when evaluating native goats, where they found low concentrations for the hormones T3 and T4 of 32 °C, 1.23 µg dL-1 and 1.97 ngmL-1, respectively, while the cortisol concentration increased at a temperature of 32 °C (6.44 ngmL-1), a behavior similar to the concentrations found for the Azul goat. Physiological variables are good indicators of animal health, but they must be properly interpreted. There is a greater classification error when considering only physiological variables, which is reduced when considering more than one

group (Correa et al., 2013), which is well considered in the equation mentioned above where we have groups of physiological and anatomical variables. C onclusions The animals had a less respiratory rate and a greater capacity of heat dissipation during dry season which allowed them to maintain rectal temperature. Morphological and hormonal (T3, T4 and cortisol) parameters change between the various climatic seasons. Rectal temperature and respiratory rate cannot purely characterize the adaptation ability of the animals. In the absence of rectal temperature changes, the animals employ erythrocyte mechanisms and increased surface temperature to dissipate heat.

Author contributions: All the authors contributed to the conception and design of the study. Neila Lidiany Ribeiro, Jaciara Ribeiro and Valquiria Cordeiro prepared the material and collected and analyzed the data. The first draft of the manuscript was written by Jaciara Ribeiro and Neila Ribeiro. Writing, proofreading and editing were carried out by Roberto Costa, Norma Ribeiro, Edilson Saraiva and Neila Ribeiro. All authors commented on previous versions of the manuscript. All authors read and approved the final manuscript. Financing information: This research did not receive external funding. Availability of data and materials: Not applicable. Code availability: Not applicable. Statements: The Animal Ethics Committee approved this study at the Federal University of Paraíba (UFPB) protocol no. 6167/18. Consent to participate: Not applicable. Consent for publication: Not applicable. Conflicts of interest: N o conflicts of interest. References B ond, T. E. , Kelly , C.F. , I ttner , N. R. 1954. Radiation studies of painted shade materials. Transaction of the ASAE, 3 5 , 389-392. B uffington , D.E. , C ollazo -A rocho , A. , C anton , G.H. , Pitt, D. 1981. Black-Globe-Humidity Index (BGHI) as comfort equations for dairy cows. St. Jo eph : ASAE , 24, 711-14. Cardoso, E.A. , Furtado, D.A. , Ribeiro, N.L. , Medeiros, A.N. , Saraiva, E.P. , Gonzaga N eto, S. , Fernandes, B.D.O. , Nascimento, J.W. 2023. Goats in a comfortable and stressed environment consuming saline water: performance, digestibility, nitrogen balance, and urinary mineral concentrations. Arquivo Brasileiro de Medicina Veterinária e Zootecnia, 75, 967-974. Cardoso, E.A. , Furtado, D.A. , Ribeiro, N.L. , Medeiros, A.N. , Saraiva, E.P. , Nascimento, J.W.B. , Sousa, F.A. , Pereira, P.H.B. 2021a. Biochemical and hormonal parameters of goats kept in a controlled environment consuming water with different levels of salinity. Arquivo Brasileiro de Medicina Veterinária e Zootecnia, 73, 853-860 Cardoso, E.A. , Furtado, D.A. , Ribeiro, N.L. , Medeiros, A.N. , Saraiva, E.P. , Nascimento, J.W.B. , Sousa, F.A. , Pereira, P.H.B. 2021b. Intake salinity water by creole goats in a controlled environment: ingestivo behavior and physiological variables. Tropical Animal Health and Production, 53, 333 E smay , M. L. 1969. Principles of animal environment , 2ed. Wastport. CT: A BI Publishing . Matos Júnior, J.L. , Furtado, D.A. , Ribeiro, N.L. , Medeiros, G.R. , Lopes Neto, J.P. , Leite, P.G. , Rodrigues, L.R. , Miranda, J.R. 2021. Salinity-water levels on productive performance, physiological and behavioral responses of 1⁄2 Dorper x 1⁄2 Santa Ines crossbred sheep. Semina: Ciências Agrárias, 42,1825-1836. P iccione , G. , R efinetti , R. 2003. Thermal chronobiology of domestic animals. Frontiers Bioscience. 1, 258-264. S ilanikove , N. 2000. Effects of heat stress on the welfare of extensively managed domestic Ruminants. Livestock Production Science, 67 , 1-18 . U do , H. M. J. 1978. Hair coat characteristics in Friesian heifers in the Netherlands and Kenya. H. Veenman e Zonen, B.V. (Eds.). (Meded. Landbouwhuge school. Wageningen) . Figure 1. Temperature values (AT), relative humidity (RH), black globe temperature (BGT) and black globe temperature and humidity index (BGHI) in the rainy and dry season Table 1. Mean±standard deviation of physiological variables in the dry and rainy seasons in the morning and afternoon of goats of the Azul genetic group Season Period R ectal temperature (°C) R espiratory rate (mov min -1) H eart rate (beat min -1) S urface temperature (°C) Rainy Morning 39.28±0.36 a 27.16±6.49 d 78.47±11.50 d 35.49±0.34 d Afternoon 39.54±0.40 a 36.50±5.09 c 83.25±15.66 c 37.49±1.25 c M ean 39.41±0.40 A 31.83±7.80 B 80.86±13.00 B 36.49±1.5 B Dry Morning 39.32±0.56 a 38.95±8.58 b 92.57±20.57 b 39.94±2.10 b Afternoon 39.53±0.40 a 39.58±10.44 a 94.75±16.79 a 40.79±1.19 a M ean 39.43±0.60 A 39.27±10.500 A 93.68±12.00 A 40.38±2.6 A a,b ,c,d Different letters in the column differ from each other by the t-test (P<0.01). A,B Lifferent letters in the column differ from each other by the t-test (P<0.01). Tab le 2 . Mean and standard deviation of the morphological parameters of the hair of female goats of the Azul genetic group Vari able Season Rainy Dry Hair length (cm) 4.20±1.20 a 3.08±0.50 b Hair tickness (mm) 0.85±0.05 a 0.65±0.06 b Hair diameter (mm) 0.07±0.01 a 0.08±0.01 b Number of hairs (hairs /cm 2) 1799.70±207.90 1734.56±205.70 a,b Different letters in the line differ from each other by the t-test (P<0.01). Tabela 3. Mean and standard deviation of the erythrogram, blood biochemical parameters and hormonal during rainy and dry season of goats Azul in the semiarid Variables Rainy Dry Erythrogram Hct % 40.14±2.60a 24.12±6.21 b Haemoglobin g/dL 15.28±1.62a 12.00±2.80 b RBC x 10 6 mL 18.22±1.92a

15.70±3.50 b MCV f/L 24.98±2.09a 10.82±3.11 b CHCM g/dL 32.91±0.21a 30.61±0.30 b Blood biochemical Glucose mg/dL 120.18±4.80a 100.94±5.72 b Cholesterol mg/dL 190.56±29.80a 162.61±35.63 b Triglycerides mg/dL 14.25±2.96b 17.58±3.95 a Urea mg/dL 72.13±10.06b 74.10±12.24 a Creatinine mg/dL 1.20±0.29a 1.12±0.33 b Total Protein g/dL 4.68±0.65b 7.37±0.88 a Albumin g/dL 1.98±0.45b 2.86±0.49 a Globulin g/dL 2.71±0.58b 4.49±1.01 a GGT U/L 49.21±10.11a 41.61±9.14b AST U/L 82.18±14.20a 71.89±13.81b ALT U/L 70.20±15.14b 95.42±9.32a Hormonal T4 μ g/mL 1.54±0.19a 0.99±0.29b T3 μ g/mL 1.89±0.23a 1.00±0.20b Cortisol ng/mL 3.29±0.90b 5.80±1.29a a,b Different letters in the line differ from each other by the t-test (P<0.01). Tab le 4 . Eigenvalues, percentage of variance and correlations (factor loading) of each variable and its respective principal componente (PC) Vari ables PC 1 PC2 PC3 Hemat ocrit 0,930 -0,035 -0,019 Mean red blood cell volume 0,920 -0,044 -0,199 Hemoglob in 0,601 -0,283 0,098 Er ythrocytes 0,615 -0,165 0,224 Hair lenght 0,584 -0,468 0,402 Hair diameter -0,183 -0,384 0,701 Hair tickness -0,473 -0,313 0,535 Rectal temperature 0,057 -0,563 -0,486 Respiratory rate -0,198 -0,698 -0,271 Heart rate -0,265 -0,755 -0,276 Surface temperature -0,728 -0,129 -0,071 Eigenvalue 3,694 1,971 1,434 Cumulative variation 33,588 51,509 64,548 Figure 2. 3D graph of the behavior of goats from the Azul genetic group in the two seasons evaluated1

SCAN SETTINGS

These features were chosen to create this report

Omit settings

References:	Off
Quotes:	Off
Citations:	Off
Titles:	Off
HTML Templates:	Off
Table Of Contents:	Off
Code Comments:	Off

Repositories

Plagiarism Detection Settings

Security Measures

Safe Search:	Off
Hide Sensitive Data:	Off
Character Manipulation:	Off

Similarity Level

Identical:	On
Minor Changes:	On

Results Calibration

Focused Results	Sensitivity: 3

Scan id:
707a27d5-569a-4a60-9419-cc64a1ead114

Learn more about these features at:
help.copyleaks.com
